# The role of self-blame and worthlessness in the psychopathology of major depressive disorder

**DOI:** 10.1016/j.jad.2015.08.001

**Published:** 2015-11-01

**Authors:** Roland Zahn, Karen E. Lythe, Jennifer A. Gethin, Sophie Green, John F. William Deakin, Allan H. Young, Jorge Moll

**Affiliations:** a Institute of Psychiatry, Psychology & Neuroscience, King’s College London, Department of Psychological Medicine, Centre for Affective Disorders, London SE5 8AZ, UK; b The University of Manchester & Manchester Academic Health Sciences Centre, School of Psychological Sciences, Neuroscience and Aphasia Research Unit, Manchester M13 9PL, UK; c The University of Manchester & Manchester Academic Health Sciences Centre, Institute of Brain, Behaviour and Mental Health, Neuroscience & Psychiatry Unit, Manchester M13 9PL, UK; d Cognitive and Behavioral Neuroscience Unit, D’Or Institute for Research and Education (IDOR), 22280-080 Rio de Janeiro, RJ, Brazil

**Keywords:** Moral emotions, Attributional style, Major depression, Self-blame, Symptoms, Nosology

## Abstract

**Background:**

Cognitive models predict that vulnerability to major depressive disorder (MDD) is due to a bias to blame oneself for failure in a global way resulting in excessive self-blaming emotions, decreased self-worth, hopelessness and depressed mood. Clinical studies comparing the consistency and coherence of these symptoms in order to probe the predictions of the model are lacking.

**Methods:**

132 patients with remitted MDD and no relevant lifetime co-morbid axis-*I* disorders were assessed using a phenomenological psychopathology-based interview (AMDP) including novel items to assess moral emotions (*n*=94 patients) and the structured clinical interview-I for DSM-IV-TR. Cluster analysis was employed to identify symptom coherence for the most severe episode.

**Results:**

Feelings of inadequacy, depressed mood, and hopelessness emerged as the most closely co-occurring and consistent symptoms (≥90% of patients). Self-blaming emotions occurred in most patients (>80%) with self-disgust/contempt being more frequent than guilt, followed by shame. Anger or disgust towards others was experienced by only 26% of patients. 85% of patients reported feelings of inadequacy and self-blaming emotions as the most bothering symptoms compared with 10% being more distressed by negative emotions towards others.

**Limitations:**

Symptom assessment was retrospective, but this is unlikely to have biased patients towards particular emotions relative to others.

**Conclusions:**

As predicted, feelings of inadequacy and hopelessness were part of the core depressive syndrome, closely co-occurring with depressed mood. Self-blaming emotions were highly frequent and bothering but not restricted to guilt. This calls for a refined assessment of self-blaming emotions to improve the diagnosis and stratification of MDD.

## Highlights

• Feelings of inadequacy were part of the core major depressive syndrome
• Self-blaming emotions occurred in most patients
• Self-disgust/contempt was slightly more frequent than guilt
• Only a minority of patients was most distressed by negative emotions towards others
• This demonstrates a central role of self-blaming emotions in major depression

## 1 Introduction

The influential revised learned helplessness model (Abramson et al., 1978) predicts that vulnerability to major depressive disorder (MDD) is due to a bias to blame oneself for failure in an overgeneralised way resulting in decreased self-worth, hopelessness and depression. Overgeneralised self-blame is associated with excessive self-blaming moral emotions((Green et al., 2013b), e.g. guilt, shame, disgust/contempt towards oneself). This is in contrast to the most widely employed model of depression that claims an overall increase in negative and reduction in positive emotions (Watson et al., 1988).

Recent evidence using experimental probes of moral emotions in remitted MDD has pointed to a relative proneness to feeling disgust/contempt towards oneself with a reduction in disgust/contempt towards others (Green et al., 2013b; Zahn et al., 2015) in support of the revised learned helplessness model. The clinical literature, however, has provided contradictory evidence regarding the role of worthlessness and self-blaming emotions in MDD, which the model predicts to be of core pathophysiological importance.

In support of the model, the combined guilt and worthlessness item in DSM (APA, 2000) was found to be most distinctive of current MDD compared with a generalized anxiety disorder group (Breslau and Davis, 1985). Further support has been provided by the largest transcultural study on MDD, where the feeling of inadequacy (including self-worthlessness) was reported as a consistent symptom of depression (Sartorius et al., 1980). Subsequent studies, however, have reported a wide variation in the consistency of guilt/worthlessness which was most often reported as a single item following DSM. The frequency of guilt/worthlessness in current MDD was found to be between 20% in Australia (Carragher et al., 2011) and Japan (Saito et al., 2010), 50% in Benin (Bertschy et al., 1992), and 70–80% in the USA (Buchwald and Rudickdavis, 1993) and France (Corruble et al., 2009). DSM worthlessness was separately reported in another US study as being present in 61% of current MDD patients (McGlinchey et al., 2006).

The clinical assessment of self-blaming emotions has classically been restricted to guilt, which was found only in a subgroup of patients (McGlinchey et al., 2006; Sartorius et al., 1980). Although, early studies claimed transcultural variation in the frequency of guilt (Gada, 1982; Stompe et al., 2001), more recent evidence suggests that guilt is experienced in a large subgroup of patients across different cultures (Bhugra and Mastrogianni, 2004). This is contradicted by a large study reporting markedly lower frequencies of guilt in Korean compared to US patients with MDD (Jeon et al., 2014). On a cautionary note, this study used item comparisons of the Hamilton Depression scale without using semi-structured interviews to elicit the information and without reporting how items were translated and culturally adapted.

The discrepancy in reported frequencies of guilt and worthlessness is likely due to methodological as well as sampling differences. The semi-structured interviews for DSM were designed to provide reliable diagnoses rather than to assess single symptoms or the coherence of symptoms (First et al., 2002). As a consequence, the criterion threshold for different items on the DSM varies between symptoms rendering a direct comparison and analyses of symptom coherence invalid. Furthermore, the role of self-blaming emotions such as self-disgust/contempt, found to be elevated in MDD using specific instruments of assessment (Green et al., 2013b; Zahn et al., 2015), remains elusive. This is because clinical assessments have solely reported guilt or non-specific reports of self-blame.

As Jaspers, the founder of phenomenological psychopathology, noted on the analyses of symptom-complexes (Jaspers, 1963/1959, p. 582ff): There are different aspects of the relation of symptoms within a symptom-complex: (1) frequency of symptom co-occurrence, and (2) coherence of symptoms by being related to a common aspect or function. The latter aspect has been emphasized by Schneider when discussing symptoms: “Their connectedness must be due to a normal complex of psychic function, which complex has been affected by the illness”. At the time of this theory, a lack of knowledge about neurobiologically valid models of many higher cognitive functions hampered the success of this approach. Aided by advances in social cognitive neuroscience, we can now aim at isolating symptom-complexes which are likely to be associated with a restricted set of cognitive-anatomical syndromes (Zahn, 2009). The neural architecture underpinning the tendency to overgeneralize self-blaming emotions in MDD has recently been elucidated (Green et al., 2013a, 2012). This supports the neurobiological validity of self-blaming emotional biases in MDD and has prompted the current study into the phenomenology of associated clinical symptoms.

Here, we investigated the following hypotheses derived from the revised learned helplessness model: (1) The feeling of inadequacy/worthlessness is a consistent symptom of MDD and co-occurs with other core symptoms when assessed using an instrument designed to assess individual symptoms (AMDP, (Ahrens and Stieglitz, 1998; Busch et al., 1980)) rather than those used in DSM validation studies. (2) The type of self-blaming emotion experienced during depressive episodes differs between patients and is not restricted to guilt. (3) Negative emotions towards others are infrequent and do not co-occur with core depression symptoms.

## 2 Methods

### 2.1 Participants

This study was approved by the South Manchester NHS Research Ethics Committee. All participants gave written informed consent and were compensated for time and travel costs. 132 (37 male) patients with major depressive disorder (MDD), fully remitted for >6 months, were enrolled (*n*=121 medication-free at time of study) and had no current, as well as no relevant past co-morbid axis-*I* disorders (see also Supplementary Methods).

Residual symptoms were assessed using the Montgomery-Åsberg-Depression-Scale ((Montgomery and Åsberg, 1979), MADRS) and psychosocial functioning was assessed using the Global-Assessment-of-Functioning (GAF, (First et al., 2002)) Scale (Axis V, DSM-IV). Remitted MDD patients had GAF scores indicating minimal or absent symptoms and high psychosocial functioning (mean=84.4±6.6) and MADRS scores that were well below the cut-off for depression of 10 (mean=1.2±1.6). Their average age was 32.8±12.3 (range 18–65), years of education mean was 16.6±2.4 (range 11–22) and their age at onset ranged from 8 to 52 (mean=21.5±8.6, for further clinical details and cultural background see Supplementary Tables S2 & S3).

### 2.2 Psychopathological assessment

We assessed 132 patients using a phenomenological psychopathology-based instrument (AMDP) translated from German (Faehndrich and Stieglitz, 1997, 2007; Guy and Ban, 1982), adding new items to assess moral emotions (*n*=94 patients). In accordance with the SCID-I (First et al., 2002), we asked patients about the worst two weeks of their last and most severe episode. Importantly all symptoms were measured on the same 4 point scale (0=absent, 1=mild/minimal, 2=moderate, 3=severe) without pre-defining different diagnostically relevant thresholds for different symptoms as is done on the SCID-I. English translations of symptom labels correspond to published symptom label translations (Faehndrich and Stieglitz, 1997). Instructions for ratings were based on definitions in the German version. In addition to the existing standard items of feelings of inadequacy and guilt, we developed additional items to assess moral emotions more systematically (the Moral Emotion Addendum to the AMDP, see Supplementary Methods). This was based on our previous work on experimental probes of moral emotions (Green et al., 2013b; Zahn et al., 2015) and their distinct neural correlates (Green et al., 2012; Moll et al., 2007; Pulcu et al., 2014; Zahn et al., 2009). Inter-rater reliability for the AMDP and moral emotion items were very high (Supplementary Table S4).

### 2.3 Data analysis

All analyses were carried out using SPSS21 (www.spss.com) at *p*=.05, 2-sided. Symptom ratings were transformed into two categories: absent to mild (0 and 1) vs. moderate to severe (2 and 3). Hierarchical cluster analysis (binary Euclidean distance, Ward method) was employed to identify symptom coherence.

## 3 Results

### 3.1 AMDP depression items

**Fig. 1:**
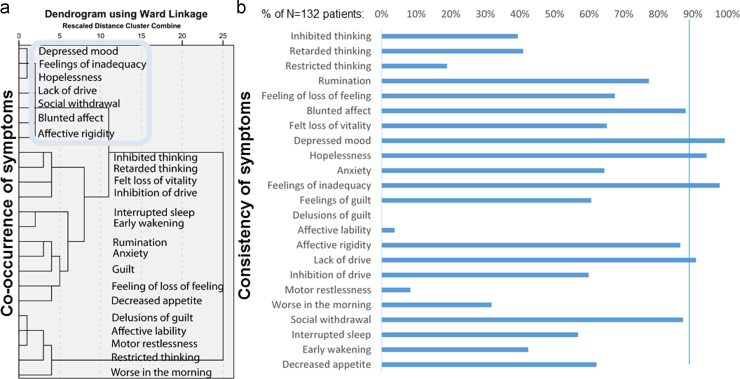
Panel (a) displays the results of the cluster analysis for depressive symptoms (of at least moderate severity). Panel (b) shows the percentage of patients reporting a particular symptom (of at least moderate severity, which was only rated when there was evidence of significant distress or impairment).

Fig. 1 shows that feelings of inadequacy (feeling worth less than others or feeling that one fails) very closely co-occurred with hopelessness and depressed mood. Together these symptoms were the most consistent, occurring in more than 90% of patients. This core cluster closely co-occurred with another cluster of symptoms of high consistency around 90%: lack of drive (this item includes lack of energy), affective rigidity (i.e. an inability to respond emotionally including anhedonia), blunted affect, and social withdrawal.

### 3.2 Moral emotions addendum

**Fig. 2:**
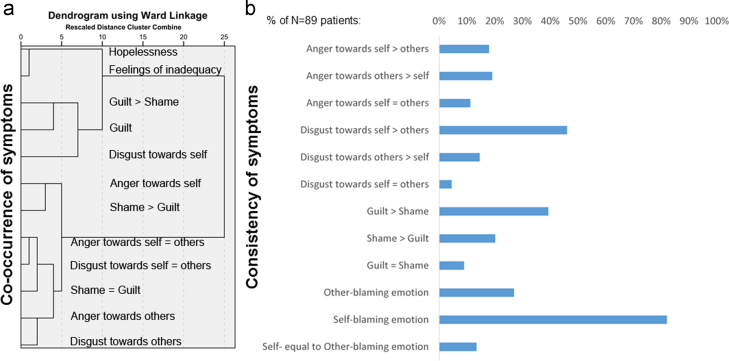
Panel (a) displays the results of the cluster analysis for the moral emotion addendum and other core depressive symptoms (of at least moderate severity). Panel (b) shows the percentage of patients reporting being bothered by a particular moral emotion (of at least moderate severity). Please note that the guilt item on the AMDP asks for self-reproach and worrying to have done something wrong. In contrast, the guilt vs. shame item on the moral emotions addendum asks whether patients have experienced guilt or shame and which was more relevant (see also Supplementary Methods). 94% of patients (65/69) reporting guilt or shame thought that these emotions can be distinguished. When asked which of the moral emotions, including feelings of inadequacy, were the most bothering, 45.8% of patients (38/83) named feelings of inadequacy as most bothering (“worthlessness” (*n*=32),“low self-confidence” (*n*=3), “low self-esteem” (*n*=2), or “low self-worth” (*n*=1)). 9.6% of patients (8/83) reported negative emotions towards others as most bothering (“anger towards others” (*n*=6), “disgust towards others” (*n*=1), “contempt towards others” (*n*=1)). “Anger” (*n*=3) and “contempt” (*n*=1) towards both self and others were even less frequent as most bothering symptoms (4.8% of patients: 4/83). Self-blaming emotions were named as most bothering by 39.8% of patients (33/83: “guilt” (*n*=11), “guilt and shame” (*n*=2), “shame” (*n*=4), “self-disgust” (*n*=1), “self-contempt” (*n*=3), “self-loathing” (*n*=6), “self-hate” (*n*=4), “self-disgust and worthlessness” (*n*=1), “anger” towards self (*n*=1)). Out of the 60 patients reporting disgust, most preferred the label “loathing” (*n*=24), followed by “hate” (*n*=16), “disgust” (*n*=12), “contempt” (*n*=7), and “worthlessness” (*n*=1).

Fig. 2 shows that self-blaming emotions occurred in most patients (82%). Interestingly, self-disgust/contempt (46%) was slightly more frequent than guilt (39%), followed by shame (20%). Anger or disgust towards others was experienced by 26% of patients. The cluster analysis revealed that guilt as measured on the AMDP and on the moral emotion addendum closely co-occurred with self-disgust/contempt and clearly separated from other moral emotions. Furthermore, the cluster comprising guilt and self-disgust/contempt occurred most closely with core depressive symptoms (hopelessness and feelings of inadequacy), whilst the other moral emotions showed only very distant relationships with core depressive symptoms.

## 4 Discussion

Consistent with the learned helplessness model, feelings of inadequacy and hopelessness were part of the core syndrome of major depression. Self-blaming emotions were very common but self-disgust/contempt was slightly more frequent than guilt. Guilt and self-disgust most closely co-occurred with core depressive symptoms, whilst negative emotions towards others were infrequent and not associated with core symptoms.

Our results are in keeping with the finding that feelings of inadequacy are a consistent feature of MDD, whilst feelings of guilt are more variable (Ebert et al., 1995; Prosen et al., 1983). This contrasts with another study reporting guilt in over 80% of MDD patients (Leckman et al., 1984) which is similar to our overall frequency for self-blaming emotions. These differences are likely due to the way guilt was assessed.

Population-based studies using the Composite International Diagnostic Interview yielded only around 20% of patients with current MDD fulfilling the DSM worthlessness/guilt criterion (Carragher et al., 2011; Saito et al., 2010). DSM-IV-TR (APA, 2000, p. 350) states that “Blaming oneself for failing to meet occupational or interpersonal responsibilities as a result of depression is very common and, unless delusional, is not considered sufficient to meet this criterion.” In practice it is very difficult to assess whether self-blame and inadequacy is felt because of being ill or because of failing generally, which explains the variation and probably underreporting of the item in DSM-based studies.

The frequency of other-blaming emotions in our study is similar to previous reports for MDD with anger attacks and hostile depression (Fava, 1998) and to the frequency of anger/irritability in current MDD (Pasquini et al., 2004). In keeping with our findings, Fava et al. (1998) observed feelings of worthlessness to be common in patients with outwardly directed anger. Unlike our study, however, studies of depression with anger attacks did not exclude patients with low psychosocial functioning outside of depressive episodes, and in fact showed an association of anger attacks with personality disorders (Fava, 1998). Patients with subsyndromal and mixed bipolar disorders, however, were not rigorously excluded from our study and may have inflated the frequency of other-blaming emotions, given the higher likelihood in these patients (Benazzi and Akiskal, 2005; Bertschy et al., 2008; Swann et al., 2007).

### 4.1 Limitations

Symptom assessment was retrospective and could have led to an underestimation of the overall frequency due to recall biases, but this is unlikely to have biased patients towards particular emotions relative to others. Assessing remitted MDD patients, however, allowed us to exclude co-morbidity with more rigor than by studying current MDD during which co-morbid personality disorders are harder to detect.

## 5 Conclusions

Feelings of inadequacy, hopelessness and self-blaming emotions are closely associated with depressed mood and are associated with high distress. This calls for sensitive probes of these symptoms in clinical assessment tools. Future studies are needed to confirm these results in patients with current MDD and to investigate how distinctive self-blaming emotions are of MDD without co-morbid personality and relevant axis-*I* disorders as studied here.

## 6 Conflict of interest

The authors have no conflict of interest to declare.

## Contributors

Jorge Moll, John F. William Deakin, and Roland Zahn designed the study. Karen Lythe, Jennifer Gethin, Sophie Green and Roland Zahn carried out the study. Karen Lythe, Jennifer Gethin and Roland Zahn analyzed the data. Roland Zahn wrote the first draft of the paper. All authors significantly contributed to and have approved the final manuscript.

## Role of the funding source

RZ was funded by a University of Manchester Stepping Stones Fellowship & an MRC clinician scientist fellowship (G0902304). SG and JG were funded by MRC and EPSRC PhD studentships respectively. The funders had no role in study design; in the collection, analysis and interpretation of data; in the writing of the report; and in the decision to submit the paper for publication.

## Acknowledgments

The project was funded by an MRC Clinician Scientist Fellowship (to RZ, G0902304), EPSRC (to JG), and MRC (to SG) PhD studentships. We are grateful to Clifford Workman for phone-screening participants and Rolf-Dieter Stieglitz for commenting on the manuscript.

## References

[bib41] Abramson L.Y., Seligman M.E.P., Teasdale J.D. (1978). Learned helplessness in humans - critique and reformulation. J. Abnorm. Psychol..

[bib1] APA (2000). Diagnostic and Statistical Manual of Mental Disorders.

[bib2] Ahrens B., Stieglitz R.D. (1998). Psychopathological assessment and diagnosis – a study of specificity of single symptoms. Psychopathology.

[bib3] Benazzi F., Akiskal H. (2005). Irritable-hostile depression: further validation as a bipolar depressive mixed state. J. Affect. Disord..

[bib4] Bertschy G., Gervasoni N., Favre S., Liberek C., Ragama-Pardos E., Aubry J.M., Gex-Fabry M., Dayer A. (2008). Frequency of dysphoria and mixed states. Psychopathology.

[bib5] Bertschy G., Viel J.F., Ahyi R.G. (1992). Depression in Benin-an assessment using the comprehensive psychopathological rating-scale and the principal component analysis. J. Affect. Disord..

[bib6] Bhugra D., Mastrogianni A. (2004). Globalisation and mental disorders – overview with relation to depression. Br. J. Psychiatry.

[bib7] Breslau N., Davis G.C. (1985). Refining DSM-III criteria in major depression-an assessment of the descriptive validity of criterion symptoms. J. Affect. Disord..

[bib8] Buchwald A.M., Rudickdavis D. (1993). The symptoms of major depression. J. Abnorm. Psychol..

[bib9] Busch H., Cranach M.V., Gulbinat W., Renfordt E., Tegeler J. (1980). Reliability of the amdp-system-a preliminary-report on a multicenter exercise on the reliability of psychopathological assessment. Acta Psychiatr. Scand..

[bib10] Carragher N., Mewton L., Slade T., Teesson M. (2011). An item response analysis of the DSM-IV criteria for major depression: findings from the Australian National Survey of Mental Health and Wellbeing. J. Affect. Disord..

[bib11] Corruble E., Chouinard V.-A., Letierce A., Gorwood P.A.P.A., Chouinard G. (2009). Is DSM-IV bereavement exclusion for major depressive episode relevant to severity and pattern of symptoms? A case-control, cross-sectional study. J. Clin. Psychiatry.

[bib12] Ebert D., Martus P., Lungershausen E. (1995). Change in symptomatology of melancholic depression over 2 decades-core symptoms and culturally determined symptoms. Psychopathology.

[bib13] Faehndrich E., Stieglitz R.D. (1997). Das AMDP-System, Manual zur Dokumentation Psychiatrischer Befunde.

[bib14] Faehndrich E., Stieglitz R.D. (2007). Leitfaden zur Erfassung des Psychopathologischen Befundes, Halbstrukturiertes Interview anhand des AMDP-Systems.

[bib15] Fava M. (1998). Depression with anger attacks. J. Clin. Psychiatry.

[bib16] First M.B., Spitzer R.L., Gibbon M., Williams J.B.W. (2002). Structured Clinical Interview for DSM-IV-TR Axis I Disorders, Research Version, Patient Edition. (SCID-I/P) Biometrics Research.

[bib17] Gada M.T. (1982). A cross-cultural-study of symptomatology of depression-eastern versus western patients. Int. J. Soc. Psychiatry.

[bib18] Green S., Lambon Ralph M.A., Moll J., Zakrzewski J., Deakin J.F., Grafman J., Zahn R. (2013). The neural basis of conceptual-emotional integration and its role in major depressive disorder. Soc. Neurosci..

[bib19] Green S., Moll J., Deakin J.F.W., Hulleman J., Zahn R. (2013). Proneness to decreased negative emotions in major depressive disorder when blaming others rather than oneself. Psychopathology.

[bib20] Green S., Ralph M.A.L., Moll J., Deakin J.F.W., Zahn R. (2012). Guilt-selective functional disconnection of anterior temporal and subgenual cortices in major depressive disorder. Arch. Gen. Psychiatry.

[bib21] Guy W., Ban T.A. (1982). The AMDP-System.

[bib22] Jaspers K. (1963/1959). General Psychopathology.

[bib23] Jeon H.J., Walker R.S., Inamori A., Hong J.P., Cho M.J., Baer L., Clain A., Fava M., Mischoulon D. (2014). Differences in depressive symptoms between Korean and American outpatients with major depressive disorder. Int. Clin. Psychopharmacol..

[bib24] Leckman J.F., Caruso K.A., Prusoff B.A., Weissman M.M., Merikangas K.R., Pauls D.L. (1984). Appetite disturbance and excessive guilt in major depression-use of family study data to define depressive subtypes. Arch. Gen. Psychiatry.

[bib25] McGlinchey J.B., Zimmerman M., Young D., Chelminski I. (2006). Diagnosing major depressive disorder VIII-Are some symptoms better than others?. J. Nerv. Ment. Dis..

[bib26] Moll J., de Oliveira-Souza R., Garrido G.G., Bramati I.E., Caparelli-Daquer E.M.A., Paiva M.L.M.F., Zahn R., Grafman J. (2007). The self as a moral agent: linking the neural bases of social agency and moral sensitivity. Soc. Neurosci..

[bib27] Montgomery S.A., Åsberg M. (1979). A new depression scale designed to be sensitive to change. Br. J. Psychiatry.

[bib28] Pasquini M., Picardi A., Biondi M., Gaetano P., Morosini P. (2004). Relevance of anger and irritability in outpatients with major depressive disorder. Psychopathology.

[bib29] Prosen M., Clark D.C., Harrow M., Fawcett J. (1983). Guilt and conscience in major depressive disorders. Am. J. Psychiatry.

[bib30] Pulcu E., Lythe K., Elliott R., Green S., Moll J., Deakin J.F., Zahn R. (2014). Increased amygdala response to shame in remitted major depressive disorder. PLoS One.

[bib31] Saito M., Iwata N., Kawakami N., Matsuyama Y., Ono Y., Nakane Y., Nakamura Y., Tachimori H., Uda H., Nakane H., Watanabe M., Naganuma Y., Furukawa T.A., Hata Y., Kobayashi M., Miyake Y., Takeshima T., Kikkawa T., World Mental Hlth J. (2010). Evaluation of the DSM-IV and ICD-10 criteria for depressive disorders in a community population in Japan using item response theory.. Int. J. Methods Psychiatr. Res..

[bib32] Sartorius N., Jablensky A., Gulbinat W., Ernberg G. (1980). WHO collaborative study-assessment of depressive disorders. Psychol. Med..

[bib33] Stompe T., Ortwein-Swoboda G., Chaudhry H.R., Friedmann A., Wenzel T., Schanda H. (2001). Guilt and depression: a cross-cultural comparative study. Psychopathology.

[bib34] Swann A.C., Moeller F.G., Steinberg J.L., Schneider L., Barratt E.S., Dougherty D.M. (2007). Manic symptoms and impulsivity during bipolar depressive episodes. Bipolar Disord..

[bib35] Watson D., Clark L.A., Carey G. (1988). Positive and negative affectivity and their relation to anxiety and depressive–disorders. J. Abnorm. Psychol.

[bib36] Zahn R. (2009). The role of neuroimaging in translational cognitive neuroscience. Topics Magn. Reson. Imaging.

[bib37] Zahn R., Lythe K.E., Gethin J.A., Green S., Deakin J.F., Workman C., Moll J. (2015). Negative emotions towards others are diminished in remitted major depression. Eur. Psychiatry.

[bib38] Zahn R., Moll J., Paiva M., Garrido G., Kruger F., Huey E.D., Grafman J. (2009). The Neural basis of Human Social Values: Evidence from fMRI. Cerebral Cortex.

