# Supplementary material for: The role of self-blame and worthlessness in the psychopathology of major depressive disorder
Source: J Affect Disord. 2015 Nov 1;186:337–41. doi: 10.1016/j.jad.2015.08.001 (PMC4573463; doi:10.1016/j.jad.2015.08.001)
Supplement: Supplementary file 1 — Supplementary material [file mmc1.docx]

**SUPPLEMENTARY MATERIALS**

**The role of self-blame and worthlessness**

**in the psychopathology of major depressive disorder**

Roland Zahn^1^, Karen E. Lythe^2^, Jennifer A. Gethin^2^, Sophie Green^2^, John F. William Deakin^3^, Allan H. Young^1^, Jorge Moll^4^

*^1^ Institute of Psychiatry at King’s College London, Department of Psychological Medicine, Centre for Affective Disorders, London, SE5 8AZ, UK*

*^2^ The University of Manchester & Manchester Academic Health Sciences Centre, School of Psychological Sciences, Neuroscience and Aphasia Research Unit, Manchester, M13 9PL, UK*

*^3^ The University of Manchester & Manchester Academic Health Sciences Centre, Institute of Brain, Behaviour and Mental Health, Neuroscience & Psychiatry Unit, Manchester, M13 9PL, UK*

*^4^ Cognitive and Behavioral Neuroscience Unit, D’Or Institute for Research and Education (IDOR), 22280-080 - Rio de Janeiro, RJ, Brazil*

**Supplementary Methods**

Participants were recruited using online and print advertisements as part of the MRC-funded “Development of Cognitive and Imaging Biomarkers Predicting Risk of Self-Blaming Bias and Recurrence in Major Depression” project([Zahn et al., 2015](#_ENREF_9)) inviting individuals with a history of depression and healthy individuals with no psychiatric history to participate. Initial suitability was assessed with a phone pre-screening interview (for exclusion reasons see Table 1) which included questions about personal history of major physical illnesses, substance abuse, axis-I disorders, psychological and pharmacological treatment, as well as family history of psychiatric disorders (a copy of a modified version of the previously described ([Green et al., 2012](#_ENREF_5)) phone screening interview can be obtained at http://www.translational-cognitive-neuroscience.org/start/test-materials). *Inclusion criteria were*: right handedness, English as first language, aged 18-65 years old, and at least one past major depressive (MD) episode according to Diagnostic Statistical Manual (DSM-IV-TR, American Psychiatric Association) that was a moderate to severe depressive episode according to the International Classification of Diseases (ICD-10, World Health Organization) with at least 2 month duration requiring treatment and remission of symptoms for at least 6 months.

*Exclusion criteria were*: centrally active medications other than hormonal contraceptives, residual symptoms of or manifest axis-I disorders ([DSM-IV](#_ENREF_1)), significant psychosocial impairment as an indicator of a clinically relevant personality disorder or incomplete remission, a Montgomery-Åsberg Depression Rating Scale (MADRS, ([Montgomery and Åsberg, 1979](#_ENREF_7))) score >10 (=cut-off for depression ([Zimmerman et al., 2004](#_ENREF_10))), current self-harming behaviour, a history of alcohol or substance abuse, schizophrenia, schizo-affective disorder, bipolar disorder, developmental disorders, learning disabilities, neurological illnesses or physical illnesses that significantly impair psychosocial functioning or brain function*,* depressive episodes secondary to another psychiatric disorder.

Suitable participants according to the phone pre-screening (see Supplementary Table 1) were invited for a clinical interview by a senior psychiatrist (RZ) and a structured assessment by either KL or JG after having been extensively trained in joint assessments and achieving high inter-rater reliability (KL, JG, RZ had completed the recommended SCID-I-training and inter-rater reliability was very good: κ=0.64 [KL&RZ] and κ=1 [KL&JG] for past MDD; κ=.60 [KL&RZ] and κ=1 [KL&JG] for past MDD including melancholic and atypical specifiers). Assessment of the first 28 patients varied slightly as previously described ([Green et al., 2013](#_ENREF_6)). Psychiatric, medical and family history was assessed while assuring strict confidentiality. The following instruments were administered: the Structured Clinical Interview for DSM-IV (SCID-I, ([First et al., 2002](#_ENREF_4))), which was modified to allow lifetime diagnoses of MDD subtypes and was used as a standardized measure to verify diagnosis of MDD ([DSM-IV](#_ENREF_1)), and a shortened version of the Weissman Family History Screen ([Weissman et al., 2000](#_ENREF_8)) which was used to assess the psychiatric history of first degree family members.

*Psychopathological assessment*

Depressive symptoms were selected from the semi-structured interview for the AMDP system ([Faehndrich and Stieglitz, 2007](#_ENREF_3)); they were translated with kind permission of the publisher for non-commercial use by RZ and checked by a native speaker (SG) for comprehensiveness. English translations of the symptom labels correspond to the published AMDP manual ([Faehndrich and Stieglitz, 1997](#_ENREF_2)) and instructions for rating were based on definitions there.

**MORAL EMOTION ADDENDUM TO THE AMDP**

| **Feelings of inadequacy**   - How is your self-confidence and self-esteem? - Do you think that you do not succeed in the same way as you used to? - Do you sometimes think that you are worth less than other people? | S |  | absent | mild | medium | worthless all aspects | N/A |
| --- | --- | --- | --- | --- | --- | --- | --- |
| **Feelings of anger**   - Do you sometimes feel angry? - Is this mostly towards yourself or others, or both? - What are typical situations? - How often and how much bothering? - Do you get physically aggressive or do you shout? | S | Towards Self = 1, Other = 2, Both = 3 | absent | mild | medium | Severe & not tied to typical situation | N/A |
| **Feelings of contempt/disgust**   - Do you sometimes feel disgust, contempt, hate, or loathing? - Which term would you prefer? - Is this mostly towards yourself or others, or both? - What are typical situations? - How often and how much bothering? - Do you get physically aggressive or do you shout? | S | Towards Self = 1 Other = 2 Both = 3    Preferred term: D C H L | absent | mild | medium | Severe & not tied to typical situation | N/A |
| **Feelings of shame or guilt**   - Do you sometimes feel shame or guilt? - Which is more relevant, are these the same or different? - What are typical situations? - How often and how much bothering? | S | Relevance: Guilt = 1 Shame = 2 Both(equal) = 3    Distinctiveness: 1 = Same 2 = Different | absent | mild | medium | Severe & not tied to typical situation | N/A |
| **Connection between moral feelings**   - Considering the experience of the following feelings you have mentioned (list all of the ones reported from: “low self-worth”, “guilt”, “shame”, “anger”, “contempt/disgust/hate/loathing towards self or towards others”) - Which is the one most bothering you? - What do you think is the connection between those feelings, do you think one causes the other? | S | Agree on best model with participant:  1st most bothering:    2nd most bothering: 3^rd^ most bothering:  Mark the type of connection between feelings:    -> causal one direction = 1 (e.g. 1^st^ to 2^nd^)  <- causal other direction = 2 (e.g. 2^nd^ to 1^st^)  <-> causal bidirectional = 3  ¦ ¦ independent = 4  * about equally bothering | | | | | |

S=Self-observation based. Psychopathological assessment is based on the patient’s description and the clinician’s observation but the clinician has to ask free follow-up questions to get enough detail to be able to interpret the response. N/A = not applicable, because symptom cannot be assessed with certainty. The first item ([Faehndrich and Stieglitz, 1997](#_ENREF_2)) was translated from ([Faehndrich and Stieglitz, 2007](#_ENREF_3)) with kind permission of the publisher for non-commercial use by RZ and checked by a native speaker (SG) for comprehensiveness.

**Supplementary Table 1** Exclusion reasons following the phone pre-screening interview

| **Reason for exclusion** | n |
| --- | --- |
| Current antihypertensive medications or statins | 20 |
| Current antidepressant or other centrally active medications | 55 |
| Diabetes | 4 |
| Epilepsy | 5 |
| Multiple sclerosis | 3 |
| Past cancer | 7 |
| Past stroke | 1 |
| Severe developmental disorders | 1 |
| Thyroid function problems | 19 |
| Vitamin D deficiency | 1 |
| Other psychiatric disorders than MDD | 72 |
| Substance or alcohol abuse | 32 |
| Other general medical condition | 12 |
| Family history of MDD/bipolar/schizophrenia (control group) | 35 |
| Excluded because of age-matching (control group) | 3 |
| Left-handed | 22 |
| MRI contraindications | 82 |
| Non-native English speaker | 28 |
| Out of age range | 4 |
| No reason recorded | 5 |
| Withdrawal after phone-pre-screening | 33 |
| Not meeting full screening criteria for MDD | 35 |
| Not remitted for long enough | 23 |
| Fulfilling criteria for current MDD | 21 |
| *Total excluded after phone pre-screening* | *523* |

In total, 878 people participated in the phone pre-screening interview. 220 passed this screening in the remitted MDD group and were invited for the first study day. Of these, 173 individuals pre-screened as remitted MDD participants were reachable, able and willing to be seen on the first study day after reading the participant information sheet sent to them. After the first day of the study, 41 individuals from the remitted MDD group were excluded (6 fulfilled criteria for a bipolar disorder, 1 fulfilled criteria for current generalized anxiety disorder, 6 fulfilled criteria for current social anxiety disorder, 5 did not meet full criteria for MDD, 3 had not been remitted from an episode for long enough, 4 fulfilled criteria for past substance abuse, 2 were excluded due to probable personality disorders, 5 showed residual symptoms of post-traumatic stress disorder, 1 fulfilled criteria for current MDD, and 8 did not complete the full AMDP assessment).The remaining 132 participants confirmed as remitted MDD completed the full AMDP assessment.

**Supplementary Table 2** Clinical characteristics of remitted MDD group

| ***Past MDD subtype*** |  |
| --- | --- |
| With melancholic features | 68/132 |
| With atypical features | 13/132 |
| No specific subtype | 51/132 |
| ***Number of previous MDEs*** |  |
| 1 | 42/132 |
| 2 | 39/132 |
| 3 | 25/132 |
| 4 | 10/132 |
| 5 | 8/132 |
| 6 or more | 8/132 |
| Average number of previous MDEs | 2.98 ± 0.38 (range:1-44) |
| ***Last MDE details*** |  |
| Average length of MDE (months) | 13.8 ± 1.4 (range: 0.5-96) |
| Average time in remission (months) | 28.8 ± 2.9 (range: 4-282) |
| Moderate depressive episode* | 28/132 |
| Severe depressive episode* | 103/132 |
| Severe depressive episode with psychotic symptoms* | 1/132 |
| ***Life-time axis-I co-morbidity***** |  |
| Anorexia nervosa | 5/132 |
| Bulimia nervosa | 2/132 |
| Post-traumatic stress disorder | 5/132 |
| Trichotillomania | 1/132 |
| Panic disorder | 1/132 |
| No life-time co-morbidity | 118/132 |
| ***Family history*** |  |
| First degree relative with MDD (diagnosed) | 61 |
| First degree relative with MDD (questionable) | 25 |
| No family member with history of MDD | 40 |
| First degree relative with schizophrenia or bipolar disorder | 4 |
| Unknown | 2 |

*According to ICD-10 criteria, ** All co-morbid disorders were fully remitted at time of study and none of the co-morbid disorders was a likely primary cause of the depressive episodes. MDE=major depressive episode, MDD subtype classification was based on adapting the SCID-I for DSM-IV-TR to allow lifetime assessment of subtypes. All medication-free participants had stopped antidepressant medication well before the required washout phase.

**Supplementary Table 3** Ethnicity and early second languages

| **Ethnicity** | Number of patients |
| --- | --- |
| American White | 1/132 |
| Asian British | 3/132 |
| Black British | 2/132 |
| White British | 102/132 |
| Jewish British | 1/132 |
| Malaysian British | 1/132 |
| Pakistani | 1/132 |
| Spanish / White British | 1/132 |
| Missing data | 20/132 |
| **Early second language** |  |
| Gujarati, Urdu and Arabic | 1/132 |
| Punjabi | 1/132 |
| Urdu and Arabic | 1/132 |
| Yoruba and Efik | 1/132 |
| Bengali | 1/132 |
| Irish Gaelic | 1/132 |
| Italian | 2/132 |
| Polish | 1/132 |
| None | 114/132 |
| Missing data | 9/132 |

**Supplementary Table 4** Inter-rater reliability on AMDP and Moral Emotion Addendum

|  | Binarised scores | | | Raw scores | | |
| --- | --- | --- | --- | --- | --- | --- |
| AMDP symptom | RZ & KL | RZ & JG | Mean | RZ & KL | RZ & JG | Mean |
| Inhibited thinking | 1.00 | 0.81 | 0.91 | 0.97 | 0.93 | 0.95 |
| Retarded thinking | 0.89 | 1.00 | 0.95 | 0.98 | 1.00 | 0.99 |
| Restricted thinking | 0.79 | 0.62 | 0.71 | 0.95 | 0.93 | 0.94 |
| Rumination | 1.00 | 1.00 | 1.00 | 0.94 | 0.99 | 0.97 |
| Feeling of loss of feeling | 0.83 | 1.00 | 0.92 | 0.86 | 0.97 | 0.92 |
| Blunted affect | 0.70 | 1.00 | 0.85 | 0.80 | 0.93 | 0.87 |
| Felt loss of vitality | 0.91 | 1.00 | 0.96 | 0.98 | 0.95 | 0.97 |
| Depressed mood | 22/22* | 11/11* | - | 0.83 | 1.00 | 0.92 |
| Hopelessness | 21/22* | 11/11* | - | 0.95 | 0.89 | 0.92 |
| Anxiety | 0.90 | 1.00 | 0.95 | 0.99 | 0.98 | 0.99 |
| Feelings of inadequacy | 22/22* | 11/11* | - | 0.93 | 1.00 | 0.97 |
| Feelings of guilt | 0.90 | 0.62 | 0.76 | 0.96 | 0.82 | 0.89 |
| Feelings of anger | 1.00 | 1.00 | 1.00 | 1.00 | 1.00 | 1.00 |
| Feelings of contempt/disgust | 0.77 | 1.00 | 0.89 | 0.95 | 1.00 | 0.98 |
| Feelings of shame or guilt | 1.00 | 1.00 | 1.00 | 1.00 | 1.00 | 1.00 |
| Delusions of guilt | 22/22* | 10/11* | - | 22/22* | 10/11* | - |
| Affective lability | 0.65 | 1.00 | 0.83 | 0.96 | 1.00 | 0.98 |
| Affective rigidity | 0.89 | 11/11* | 0.89 | 0.95 | 1.00 | 0.98 |
| Lack of drive | 0.61 | 11/11* | 0.61 | 0.89 | 0.79 | 0.84 |
| Inhibition of drive | 0.91 | 1.00 | 0.96 | 0.97 | 0.99 | 0.98 |
| Motor restlessness | 0.65 | 1.00 | 0.83 | 0.87 | 1.00 | 0.94 |
| Worse in the morning | 0.88 | 0.62 | 0.75 | 0.96 | 0.92 | 0.94 |
| Social withdrawal | 0.54 | 11/11* | 0.54 | 0.89 | 0.65 | 0.77 |
| Interrupted sleep | 0.80 | 0.79 | 0.80 | 0.95 | 0.98 | 0.97 |
| Early wakening | 0.82 | 1.00 | 0.91 | 0.97 | 0.99 | 0.98 |
| Decreased appetite | 1.00 | 0.82 | 0.91 | 0.98 | 0.96 | 0.97 |

Of the 132 patients, 127 were primarily rated by RZ, 25 by KL and 12 by JG. 89 of RZ’s and all of KL and JG’s ratings included the moral emotion addendum. Inter-rater reliability scores are given for the binarised scores (absent/mild vs. moderate/severe) used in the hierarchical cluster analysis and raw scores (absent, mild, moderate, severe). Reliability for binarised scores is given as a kappa value and for raw scores is given as an intraclass correlation value (two-way mixed with absolute agreement). Asterisks indicate that one or both raters in the pair rated that item consistently across participants, and so an inter-rater reliability score could not be calculated; instead the proportion of agreement was listed: number of participants agreed/total number of participants rated.

**Supplementary References**

DSM-IV, American Psychiatric Association 2000. Diagnostic and Statistical Manual of Mental Disorders, Fourth Edition - Text Revision (DSMIV-TR).

Faehndrich, E., Stieglitz, R.D., 1997. Das AMDP-System, Manual zur Dokumentation psychiatrischer Befunde, 6th ed. Hogrefe Verlag, Goettingen.

Faehndrich, E., Stieglitz, R.D., 2007. Leitfaden zur Erfassung des psychopathologischen Befundes, Halbstrukturiertes Interview anhand des AMDP-Systems, 3rd ed. Hogrefe Verlag, Goettingen.

First, M.B., Spitzer, R.L., Gibbon, M., Williams, J.B.W., 2002. Structured Clinical Interview for DSM-IV-TR Axis I Disorders, Research Version, Patient Edition. (SCID-I/P) Biometrics Research, New York State Psychiatric Institute, New York.

Green, S., Lambon Ralph, M.A., Moll, J., Deakin, J.F., Zahn, R., 2012. Guilt-selective functional disconnection of anterior temporal and subgenual cortices in major depressive disorder. Arch Gen Psychiatry 69, 1014-1021.

Green, S., Moll, J., Deakin, J.F.W., Hulleman, J., Zahn, R., 2013. Proneness to Decreased Negative Emotions in Major Depressive Disorder when Blaming Others rather than Oneself. Psychopathology 46, 34-44.

Montgomery, S.A., Åsberg, M., 1979. A new depression scale designed to be sensitive to change. British Journal of Psychiatry 134, 382-389.

Weissman, M.M., Wickramaratne, P., Adams, P., Wolk, S., Verdeli, H., Olfson, M., 2000. Brief screening for family psychiatric history - The family history screen. Arch. Gen. Psychiatry 57, 675-682.

Zahn, R., Lythe, K.E., Gethin, J.A., Green, S., Deakin, J.F., Workman, C., Moll, J., 2015. Negative emotions towards others are diminished in remitted major depression. European psychiatry : the journal of the Association of European Psychiatrists.

Zimmerman, M., Posternak, M.A., Chelminski, I., 2004. Derivation of a definition of remission on the Montgomery–Asberg depression rating scale corresponding to the definition of remission on the Hamilton rating scale for depression. Journal of Psychiatric Research 38, 577-582.
